# Molecular Frontiers in Melanoma: Pathogenesis, Diagnosis, and Therapeutic Advances

**DOI:** 10.3390/ijms25052984

**Published:** 2024-03-04

**Authors:** Hyun Jee Kim, Yeong Ho Kim

**Affiliations:** 1 Department of Dermatology, International St. Mary’s Hospital, College of Medicine, Catholic Kwandong University, Incheon 22711, Republic of Korea; hyunjee0921@hanmail.net; 2 Department of Dermatology, Seoul St. Mary’s Hospital, College of Medicine, The Catholic University of Korea, Seoul 06591, Republic of Korea

**Keywords:** melanoma, therapeutics, biomarkers

## Abstract

Melanoma, a highly aggressive skin cancer, is characterized by rapid progression and high mortality. Recent advances in molecular pathogenesis have shed light on genetic and epigenetic changes that drive melanoma development. This review provides an overview of these developments, focusing on molecular mechanisms in melanoma genesis. It highlights how mutations, particularly in the *BRAF*, *NRAS*, *c-KIT*, and *GNAQ/GNA11* genes, affect critical signaling pathways. The evolution of diagnostic techniques, such as genomics, transcriptomics, liquid biopsies, and molecular biomarkers for early detection and prognosis, is also discussed. The therapeutic landscape has transformed with targeted therapies and immunotherapies, improving patient outcomes. This paper examines the efficacy, challenges, and prospects of these treatments, including recent clinical trials and emerging strategies. The potential of novel treatment strategies, including neoantigen vaccines, adoptive cell transfer, microbiome interactions, and nanoparticle-based combination therapy, is explored. These advances emphasize the challenges of therapy resistance and the importance of personalized medicine. This review underlines the necessity for evidence-based therapy selection in managing the increasing global incidence of melanoma.

## 1. Introduction

Melanoma, the deadliest form of skin cancer, presents a significant clinical challenge due to its high metastatic potential and resistance to conventional therapies. It originates from melanocytes and is increasingly prevalent [1]. 

Melanoma’s incidence surged by 320% from 1975 to 2018, influenced by risk factors such as sun exposure, indoor tanning, family history, and the number of nevi [2]. This type of skin cancer is responsible for nearly 90% of skin cancer deaths, despite constituting a small fraction of skin cancer cases [3]. The disease impacts both older and younger populations, though the increase in incidence is particularly notable among older individuals [3]. The primary cause of death from melanoma is metastatic spread, first to the lymph nodes and, most commonly, to the lungs [3]. Early-stage melanoma (I–II) can be effectively treated with complete surgical excision, boasting an excellent 5-year survival rate of 99.4% [2]. However, the prognosis worsens significantly in advanced stages, with 5-year survival rates dropping to 68% for stage III and 29.8% for stage IV melanoma [2].

The clinical burden of melanoma is growing alongside its rising global incidence, which has been increasing by approximately 3% annually in certain regions [4]. Projections for 2023 estimate 97,620 new cases and 7990 deaths in the United States alone [4]. Meanwhile, the International Agency for Research on Cancer reported an estimated 324,635 new diagnoses and 57,043 deaths from melanoma globally in 2020 [4]. These statistics highlight the critical need for the rational and evidence-based selection of therapies in the treatment of melanoma.

This review focuses on the molecular pathogenesis of melanoma, underlining recent advances in understanding the disease’s genetic and epigenetic landscape. Key genetic drivers central to melanoma development, such as mutations in the *BRAF*, *NRAS*, and *c-KIT* genes, are discussed alongside the latest WHO classification, which categorizes melanomas based on cumulative sun damage (CSD), which correlates with specific molecular alterations. This classification aids in understanding the disease’s molecular diversity [5].

Advances in genomic technologies, especially single-cell sequencing, have significantly improved the characterization of melanoma’s gene signatures and phenotypic subtypes, which are crucial for understanding its aggressiveness and high mortality rate [6]. 

Despite the high survival rate for localized melanoma, the prognosis for metastatic melanoma remains poor, highlighting the need for effective therapies [2]. The introduction of ipilimumab in 2011 marked a turning point in melanoma treatment [4]. Since then, numerous new drugs for unresectable melanoma have been approved, transforming the therapeutic landscape and improving overall survival (OS) times. This review consolidates recent findings in molecular pathology, diagnostics, and therapeutic strategies for melanoma, emphasizing the challenges of therapy resistance and the potential of personalized medicine and evidence-based therapy selection. Those approaches are essential, given the increasing global incidence and clinical burden of melanoma.

## 2. Molecular Pathology of Melanoma

The progression from benign melanocytic nevi to malignant melanoma and metastasis involves an interplay of genetic factors and UV-induced damage. Melanocytic nevi, typically benign, can evolve into melanoma through mutations, primarily *BRAF*V600E in common nevi and various mutations in MAPK signaling, the TERT promoter, and CDKN2A in dysplastic nevi [7]. The progression is marked by additional mutations, such as in NRAS, and is influenced by the activation of the WNT signaling pathway, which is important for metastasis. Genes such as *ARID2* and *ARID1A* are also implicated in melanoma’s progression [7].

### 2.1. WHO Classification and Molecular Diversity of Melanoma

The 2018 WHO classification of melanocytic lesions provides a refined understanding of melanoma’s molecular diversity, categorizing melanomas based on CSD. This system divides melanomas into:

1. Low-CSD melanomas: these include superficial spreading melanomas, typically associated with less sun damage. They often arise on the trunk and proximal areas of the extremities and primarily feature *BRAF*V600E mutations. Other mutations in these melanomas include the *TERT* promoter and *CDKN2A*, with *PTEN* and *TP53* mutations observed in more advanced stages [5].

2. High-CSD melanomas: comprising lentigo maligna and desmoplastic melanomas, these usually develop on heavily sun-damaged skin, particularly in older individuals. They can have a high mutation load, including *NRAS*, *BRAF* non-V600E, or *NF1* mutations, and they frequently present *TERT* promoter mutations, *CDKN2A*, and occasionally *KIT* mutations. The mutation count in these melanomas increases with the degree of CSD, with desmoplastic melanomas showing the highest tumor mutation burden [5].

3. Non-CSD-associated melanomas: this category contains Spitz melanomas, acral melanomas, mucosal melanomas, melanomas arising from congenital or blue nevi, and uveal melanomas. These subtypes are typically devoid of *BRAF*, *NRAS*, and *NF1* mutations (triple wild-type), but can feature *KIT* mutations, gene amplifications, and structural rearrangements, especially of the *CCND1* gene and *SF3B1*. Acral and mucosal melanomas are biologically distinct from their cutaneous counterparts in sun-exposed areas. Spitz melanomas show tyrosine kinase or serine-threonine kinase fusions, and melanomas in blue nevi and uveal melanomas often contain *GNA11* or *GNAQ* mutations [5].

This classification not only segregates melanomas based on CSD, but also correlates the types with specific molecular alterations, providing crucial insights into the varied molecular pathways and risk factors associated with different subtypes of melanoma.

In addition to these classifications, WHO has introduced the concept of „intermediate” lesions in its latest melanocytic tumor classification, acknowledging the diagnostic challenges of melanocytic tumors. This approach shifts away from viewing melanocytic tumors as simply benign or malignant, suggesting a more nuanced understanding by providing nine categories/pathways, each marked by specific genetic drivers [5].

### 2.2. Key Genetic Mutations in Melanoma

#### 2.2.1. *BRAF* Mutations

**Figure 1:**
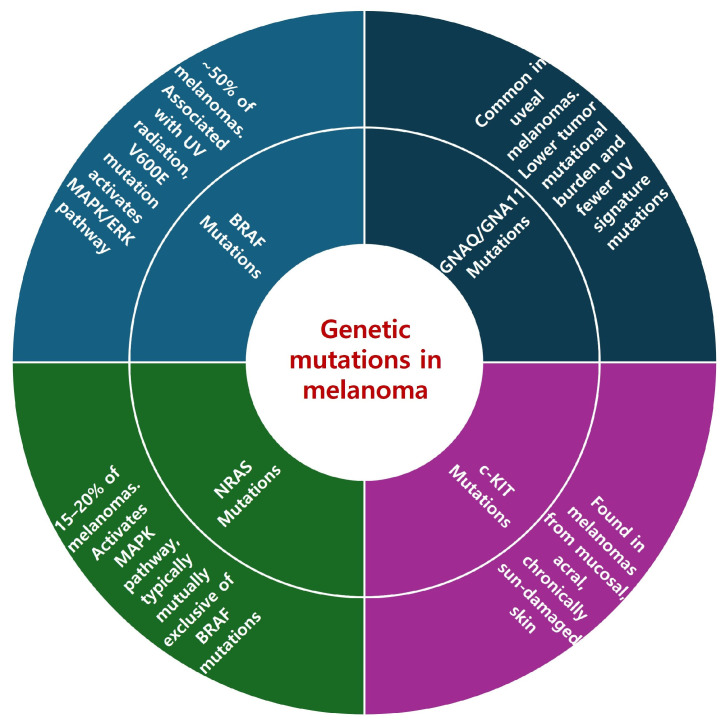
Genetic mutations in melanoma.

*BRAF* mutations are found in approximately 50% of melanomas and play a crucial role in the disease’s pathogenesis (Figure 1). These mutations are predominantly observed in cutaneous melanomas and are frequently associated with UV radiation exposure. This link is evidenced by the high levels of UV radiation signatures, especially C > T substitutions, found in these tumors [8]. Notably, patients with *BRAF* mutations tend to be younger than those with other melanoma subtypes [8]. The mutation patterns related to UV exposure highlight the interplay between environmental factors and the genetic landscape of melanoma, emphasizing the importance of considering both of them in understanding melanoma development.

The presence of *BRAF* mutations, particularly V600E, leads to the activation of the MAPK/ERK signaling pathway, a key driver of cell proliferation and survival in melanoma. This understanding has led to the development of targeted therapies such as vemurafenib and dabrafenib, which specifically inhibit the *BRAF*V600E mutation and have shown significant efficacy in treating patients harboring this genetic alteration.

In clinical practice, the detection of *BRAF* mutations is a critical factor in treatment decisions, particularly for metastatic melanoma. Testing for the *BRAF*V600 mutation is recommended for patients with distant metastases, non-resectable regional metastases, or high-risk stage III melanoma post-surgery [9]. Performing this test on metastatic tissue samples is ideal, due to the high concordance of *BRAF* mutation status in primary and metastatic lesions. The result of this testing significantly informs therapeutic decision-making, underscoring the role of *gene*tic profiling in the personalized treatment of melanoma.

#### 2.2.2. NRAS Mutations

*NRAS* mutations, which are found in 15–20% of melanoma cases, are significant drivers of the disease, affecting melanoma development through a distinct pathway [9]. These mutations activate the MAPK pathway, albeit through a mechanism different from that of *BRAF* mutations. *NRAS* mutations are typically mutually exclusive of *BRAF* mutations, reinforcing their unique role in melanoma’s molecular pathology [9]. The identification of *NRAS* mutations is not only essential for understanding melanoma’s genetic diversity, but also plays an important role in clinical decision-making, particularly in cases without *BRAF* mutations. Despite not being direct targets of current therapies, the presence of *NRAS* mutations guides the selection and tailoring of treatment strategies. So far, targeted therapies specifically addressing *NRAS*-mutated melanoma have shown limited success [5]. However, this area remains a significant focus of ongoing research, especially in the context of advanced melanoma that has not responded to standard immunotherapies, including anti-CTLA4 and anti-PD1 antibodies. The development of more effective treatment options for *NRAS*-mutated melanoma is a key objective of current clinical investigations, reflecting the continuous effort to improve therapeutic outcomes for this challenging subset of melanoma patients.

##### 2.2.3. *c-KIT* Mutations

*c-KIT* mutations, while less common than *BRAF* and *NRAS* mutations, play a significant role in certain melanoma subtypes, particularly mucosal and acral melanomas. These mutations are predominantly found in melanomas arising from mucosal, acral, and chronically sun-damaged skin, representing a distinct subset within the broader spectrum of melanoma [9].

In the clinical setting, it is recommended to initially test for *BRAF* and *NRAS* mutations in acral and mucosal melanomas [5]. If those tests return negative, a further analysis for *c-KIT* mutations is advised [5]. This stepwise approach to *gene*tic testing ensures a precise and targeted strategy for managing these specific melanoma subtypes.

Therapies targeting *c-KIT* mutations, although not yet formally approved, have shown promising results in treating melanomas harboring these genetic alterations. Clinical benefits from c-KIT inhibitors have been observed in selected patients, underscoring the importance of these mutations in the therapeutic landscape of melanoma [9]. The ongoing research and development of treatments targeting *c-KIT* mutations are pivotal in enhancing care and outcomes for patients with these specific melanoma subtypes.

#### 2.2.4. *GNAQ/GNA11* Mutations

*GNAQ/GNA11* mutations, commonly identified in uveal melanomas [10], represent a distinct genetic subgroup within melanoma. Despite the current limitations of available treatments, these mutations are under active study for targeted therapy options, reflecting a growing interest in developing specific treatments for these subtypes. 

In non-uveal melanomas, *GNAQ/GNA11* mutations display a unique genetic profile characterized by a lower tumor mutational burden and fewer UV signature mutations than are common in cutaneous melanomas [11]. This suggests significant differences in the genetic landscape of these tumors compared with both cutaneous and uveal melanomas. Additionally, non-uveal melanomas with *GNAQ/GNA11* mutations tend to metastasize lymphatically, similar to cutaneous melanoma, rather than the hematogenous metastasis typically seen in uveal melanoma [11]. These findings underline the urgent need for novel therapeutic approaches because non-uveal melanomas with *GNAQ/GNA11* mutations respond poorly to existing systemic therapies, including immune checkpoint inhibitors (ICIs) [11]. The rarity and distinct behavior of *GNAQ/GNA11* mutant non-uveal melanomas highlight the importance of ongoing research to develop effective treatments for this unique melanoma subgroup.

### 2.3. Molecular Pathways

#### 2.3.1. MAPK/ERK Pathway

The MAPK/ERK pathway is central to melanoma, with alterations in this pathway often driving tumorigenesis [9]. This pathway, which includes RAS, RAF, ERK, and mitogen-activated extracellular signal-regulated kinase (MEK), is crucial for regulating cellular proliferation [2]. The discovery of activating *NRAS* mutations in melanoma in the mid-1980s and the subsequent identification of *BRAF* mutations in 2002 have been significant milestones in understanding melanoma’s molecular pathology [12]. Both of these mutations lead to the overactivation of the MAPK/ERK pathway, promoting melanoma cell proliferation and survival. 

The exploration of the MAP kinase pathway as a therapeutic target began with responses observed from MEK inhibitors. However, the development of potent and selective inhibitors such as vemurafenib, dabrafenib, and encorafenib, which target mutated BRAF, has been the major breakthrough in melanoma treatment [12]. The late 2000s saw a pivotal shift with the introduction of these targeted therapies. The initial use of drugs such as sorafenib paved the way for more effective and selective BRAF inhibitors [12].

Combination therapies of BRAF/MEK inhibitors have significantly improved the efficacy of melanoma treatments, with reduced toxicity and longer progression-free survival (PFS) times compared with monotherapies [12]. These advances highlight the significance of the MAPK/ERK pathway in melanoma’s molecular pathology and response to therapy, underscoring the importance of understanding the influence of these mutations when developing effective treatments. 

#### 2.3.2. PI3K/AKT/mTOR Pathway

The PI3K/AKT/mTOR pathway plays a central role in melanoma development, affecting cell survival, proliferation, and metastasis. Its activation, often driven by genetic mutations and signaling imbalances, underscores the need for targeted therapeutic interventions. The constitutive activation of this pathway is a hallmark of melanoma’s aggressiveness and contributes to resistance against standard treatments by influencing key cellular processes such as autophagic cell death and cell cycle regulation [13].

A range of targeted therapies focusing on inhibitors of PI3K, AKT, and mTOR is under investigation and showing promising results in clinical trials, both as individual treatments and in combination with other drugs, such as BRAF and MEK inhibitors [13]. The exploration of natural compounds, repurposed drugs, and novel synthetic molecules targeting this pathway, along with the emerging role of miRNA in modulating this pathway, presents new opportunities for treatment and underscores the pathway’s significance for the development of novel, effective, and personalized melanoma therapies [13].

### 2.4. Melanogenesis and Neuroendocrine Regulation in Melanoma Progression

Recent studies have highlighted the complex roles of melanin and melanogenesis in melanoma, revealing their protective effects against UV radiation and their potential to promote malignant transformation [14]. The synthesis of eumelanin and pheomelanin, influenced by environmental and hormonal factors, offers both defense and risks, with the instability of pheomelanin contributing to a mutagenic environment [15,16,17,18,19,20,21,22,23,24,25]. This duality affects melanoma’s development and response to treatments, with advanced melanomas showing a negative correlation between pigmentation and survival, indicating melanin’s dual impact [26,27,28,29]. Inhibiting melanogenesis could, therefore, enhance therapeutic outcomes, highlighting melanin’s intricate influence on melanoma behavior. 

Moreover, melanoma’s ability to influence both local and systemic physiological responses through the secretion of neuroendocrine factors, including proopiomelanocortin (POMC) peptides, corticotropin-releasing hormone (CRH), and glucocorticoids, underscores its complex role in the body’s regulatory systems [30]. POMC peptides, including melanocyte-stimulating hormone (MSH), are immunosuppressive, and an increased expression of POMC peptides was noted during the progression of melanomas to advanced stages [31,32,33,34,35,36,37,38,39]. This capability of melanoma to manipulate the neuroendocrine and immune responses contributes to its survival and progression, altering homeostasis in favor of the tumor [30]. Such interactions necessitate the exploration of therapeutic strategies aimed at targeting these pathways, offering potential to improve treatment outcomes and patient prognosis. 

## 3. Molecular Diagnostic Techniques

### 3.1. Latest Molecular Diagnostic Methods

Molecular diagnostics for melanoma have made significant strides, largely due to advances in genomics, transcriptomics, and emerging techniques such as liquid biopsies.

Genomic analyses, particularly next-generation sequencing (NGS), have been essential in identifying specific mutations and genomic alterations in melanoma. NGS allows for the comprehensive screening of multiple genes associated with melanoma in a single experiment, enabling the efficient identification of mutations in key genes such as *BRAF*, *NRAS*, and *c-KIT* [9]. Although NGS is currently more prevalent as a research tool, its importance in the diagnostic setting is expected to increase, especially as more actionable mutations are identified and targeted therapies become available.

Recent developments in single-cell transcriptomics have made it possible to analyze the tumor microenvironment in melanoma in detail. This technique elucidates cellular behaviors and interactions within tumors, clarifying melanoma progression and suggesting how it might respond to treatments. By identifying various cellular subtypes and their transcriptional states, it enhances our understanding of tumor heterogeneity, which is useful in assessing treatment responses, especially in the advanced stages of melanoma [40]. Furthermore, this approach helps explain the mechanisms of response and resistance to therapies such as ICIs [40].

Additionally, liquid biopsies are emerging as a valuable non-invasive diagnostic tool. They detect circulating tumor DNA in the blood and offer insights into tumor genetics. This minimally invasive method can monitor treatment responses and disease progression, complementing traditional diagnostic techniques [9].

### 3.2. Molecular Biomarkers Used for Diagnosing and Prognosticating Melanoma

Biomarkers in melanoma encompass a wide range of indicators, from serum proteins to genetic alterations, pathology findings, and imaging results. The use of molecular biomarkers is increasingly important in the early diagnosis, staging, and prediction of therapy responses in melanoma (Table 1). 

One well-known driver mutation is *BRAF*V600E, which is responsive to BRAF inhibitors [41,42,43]. In contrast, *NRAS* mutations, although less common, are linked to shorter survival times in stage IV melanoma [44,45,46]. Unlike *BRAF* and *NRAS* mutations, *c-KIT* mutations are not closely associated with histological subtypes or tumor stage. They are more prevalent in older patients, acral mucosal melanoma subtypes, and areas with chronic sun-induced damage [47,48,49,50,51]. Furthermore, melanomas with *neurofibromin 1* (*NF1*) mutations are associated with advanced patient age and have a poorer prognosis compared to other mutation patterns [52,53]. On the other hand, female patients with high plasma membrane calcium-transporting ATPase 4 (PMCA4) transcript levels exhibit longer PFS than other patients [54]. High PMCA4 transcript levels in cutaneous melanoma are also associated with an improved prognosis, especially following PD-1 blockade therapy [54].

The tumor mutational burden (TMB) in melanoma is gaining attention for its potential to predict the response to ICIs. A high TMB might correlate with an increased effectiveness of these treatments, suggesting a potential for enhanced tumor recognition and elimination by the immune system [55,56,57]. 

Gene expression profiling (GEP), exemplified by tests such as the 31-GEP panel Decision-Dx Melanoma™, is being used to assess melanoma recurrence and metastatic risk. These tests examine the expression patterns of selected genes in the primary tumor, contributing to informed clinical decision-making [58,59].

Recent research underscores that the gut microbiome can influence carcinogenesis by producing pro- or anti-tumor inflammatory environments. Studies in melanoma indicate that the intestinal microbiota composition affects the response to ICI therapy [60]. Greater microbiota diversity, particularly certain *Ruminococcaceae* subspecies, is associated with better anti-PD-1 therapy outcomes and increased CD8+ T cell infiltration, and specific *Bacteroidetes* species might reduce the risk of ICI-induced colitis [61].

## 4. Molecular Therapeutic Strategies

The increase in survival rates seen since the advent of BRAF-MEK inhibitors and immunotherapy signifies a major breakthrough in melanoma therapeutics. These advances have not only improved outcomes, but also reshaped approaches to molecular strategies in melanoma treatment. Additionally, the evolving role of adjuvant therapy in melanoma, particularly with respect to patient selection, underscores the growing importance of personalized medicine in optimizing therapeutic efficacy and minimizing adverse effects. Furthermore, novel approaches to treatment strategies, such as neoantigen vaccines, adoptive cell transfer, and microbiome research, are expanding the horizon of melanoma management, offering new avenues for treatment (Table 2).

### 4.1. Targeted Therapy

#### 4.1.1. BRAF Inhibitors

In the context of treating *BRAF*V600E mutation-positive melanoma, vemurafenib and dabrafenib have shown significant efficacy, leading to significant improvements in PFS and OS for patients with *BRAF*-mutated melanoma [62]. These drugs target the mutated BRAF protein, effectively disrupting the MAPK/ERK signaling pathway essential for tumor growth. Furthermore, vemurafenib, the first BRAF inhibitor approved by the FDA in 2011 for metastatic melanoma with the *BRAF*V600E mutation, has demonstrated an increase in OS compared with dacarbazine in a phase III (BRIM-3) trial [63,64,65,66]. 

#### 4.1.2. MEK Inhibitors

MEK inhibitors, which target elements downstream of BRAF in the MAPK signaling pathway, are an important therapeutic option for melanoma treatment. Trametinib, which specifically targets MEK1 and MEK2, has been approved to treat metastatic or unresectable melanoma with *BRAF* V600E or V600K mutations and as adjuvant therapy [67]. The METRIC study highlighted trametinib’s effectiveness, compared with chemotherapy, in metastatic melanoma, as well as in combination with dabrafenib in brain metastases or adjuvant therapy (COMBI-d and COMBI-AD studies, respectively) [68,69,70,71]. 

#### 4.1.3. BRAF-MEK Combination Therapy

Combination therapy to block multiple points in the MAPK pathway for melanoma treatment is designed to reduce the risk of resistance development. Clinical trials such as coBRIM and COMBI-d have shown enhanced efficacy with this approach, with increased PFS and a higher overall response rate compared with monotherapy [72,73,74,75,76]. The combination of BRAF and MEK inhibitors (vemurafenib and cobimetinib) was approved in 2015 for treatment of patients with unresectable or metastatic melanoma who harbor a *BRAF* V600E or V600K mutation [77].

### 4.2. Immunotherapy

Advances in understanding the molecular pathways involved in melanoma have led to the development of successful immunotherapies for unresectable stage III and IV melanoma. These include ICIs that target PD-1 and CTLA-4, which have revolutionized treatment and prognosis in the past decade [5].

#### 4.2.1. Checkpoint Inhibitors

Both CTLA-4 and PD-1 inhibitors are the two types of checkpoint inhibitors currently available to melanoma patients. Ipilimumab, a CTLA-4 inhibitor, enhances T-cell activity by inhibiting the immunosuppressive interaction between CTLA-4 and B7 [2]. Since its approval in 2011, this immunotherapy has been associated with improved OS rates, particularly when used in conjunction with dacarbazine [78].

PD-1 inhibitors (pembrolizumab and nivolumab) are currently approved for the treatment of metastatic or unresectable melanoma and as adjuvant therapy [67]. These drugs enhance the immune system’s ability to recognize and attack cancer cells by blocking the PD-1 receptor on T cells [67]. Pembrolizumab has been reported to surpass the performance of ipilimumab for the treatment of untreated metastatic or unresectable melanoma (KEYNOTE-006 study) and chemotherapy in patients with previously treated metastatic or unresectable disease (KEYNOTE-002 study) [79,80]. Nivolumab has shown effectiveness both as a monotherapy and in combination with ipilimumab, as evidenced in the CHECKMATE-037, CHECKMATE-066, and CHECKMATE-067 studies [81,82,83,84]. The CHECKMATE-067 trial demonstrated that the combination of CTLA-4 and PD-1 inhibitors (ipilimumab and nivolumab) offers a significant survival advantage in advanced melanoma patients [84,85,86]. This combination strategy enhances the overall response rates and PFS by targeting multiple points in the immune response cascade. Although it enhanced survival outcomes, it is important to note that the combination can also increase the occurrence of adverse effects.

#### 4.2.2. Talimogene Laherparepvec (T-VEC) 

T-VEC (IMLYGIC®), the first viral oncolytic immunotherapy approved for unresectable metastatic stage IIIB/C–IVM1a melanoma, represents a notable advance in melanoma treatment [87]. This genetically modified herpes simplex type 1 virus is directly injected into tumors, triggering both local and systemic immune responses that lead to tumor cell destruction and the activation of tumor-specific T cells [88].

Clinical trials have demonstrated T-VEC’s effectiveness both as a monotherapy [89,90,91] and in combination with ICIs such as ipilimumab and pembrolizumab [92,93,94], showcasing its ability to enhance local and systemic anti-tumor responses. T-VEC has shown promising efficacy and tolerable side effects in treating both injected and non-injected melanoma lesions, although its systemic effect as a single-agent therapy is relatively modest [89,90,91]. T-VEC’s innovative method for inducing a comprehensive immune response against melanoma cells, along with its synergistic potential with other immunotherapies, makes it a valuable melanoma treatment option.

### 4.3. Combination of Targeted Therapy and Immunotherapy

Current research efforts are focusing on integrating BRAF and MEK inhibitors, types of targeted therapies, with immunotherapy agents. The rationale behind this strategy is to combine the direct action of the targeted therapy, which addresses specific genetic mutations in melanoma cells, with the broad-acting capacity of immunotherapy to bolster the immune system’s cancer-fighting abilities. The goal is to potentially increase treatment effectiveness and counteract drug resistance. 

One example of such research is a clinical trial investigating the combination of dabrafenib (a BRAF inhibitor) and trametinib (a MEK inhibitor) with pembrolizumab, an anti-PD-1 therapy [95,96]. This trial is particularly focused on evaluating the synergistic effects of these treatments in patients with advanced melanoma. Additionally, the SECOMBIT trial demonstrated that sequential immunotherapy (nivolumab plus ipilimumab) and targeted therapy (encorafenib plus binimetinib) provide clinically significant survival benefits in patients with *BRAF*-mutant melanoma [97].

### 4.4. Integration of Surgical and Systemic Therapies

The integration of surgical interventions and systemic therapies, including pre-surgical (neoadjuvant) and post-surgical (adjuvant) treatments, is an area of active research. This multidisciplinary approach aims to improve OS rates, reduce recurrence, and manage metastatic disease more effectively.

Adjuvant therapy plays a crucial role in managing melanoma, affecting both OS and recurrence-free survival times. Although interferon-α was previously a common choice, it has been replaced by safer and more effective options, such as ICIs and targeted therapies. Patients with lymph node involvement often receive BRAF/MEK inhibitors if they have the *BRAF*V600 E/K mutation. Anti-PD-1 therapies, including pembrolizumab, are also an option regardless of mutation status [67].

Pembrolizumab is FDA-approved as adjuvant therapy for the treatment of stage IIB/IIC and III melanoma [98]. The KEYNOTE-716 trial demonstrated that pembrolizumab has a significant and durable impact on distant-metastasis-free survival and recurrence-free survival in resected stage IIB or IIC melanoma patients [99]. Moreover, the KEYNOTE-942 trial introduced a combination of the investigational mRNA-4157/V940 vaccine and pembrolizumab and found that it shows potential in reducing the risk of disease recurrence after surgery [100].

In addition, Schumann K and colleagues have shown the effectiveness of other adjuvant therapies, such as nivolumab and the combination of dabrafenib and trametinib (D + T), in a broader range of patients than is typically included in clinical trials [101]. This suggests the applicability of clinical trial results to everyday clinical practice and highlights the importance of evaluating these therapies in diverse patient groups.

Additionally, the CHECKMATE-238 trial is examining the long-term effects of adjuvant nivolumab compared with ipilimumab in patients with resected stage III/IV melanoma [102]. This contributes to the growing understanding of effective adjuvant treatments in melanoma.

### 4.5. Novel Approaches to Treatment Strategies

#### 4.5.1. Neoantigen Vaccines

Renewed interest in cancer vaccines, particularly for melanoma, has focused on personalized neoantigen vaccines. These vaccines are tailored to individual patients and target unique tumor-specific antigens that arise from mutations. Clinical trials of neoantigen vaccines in melanoma have shown them to be safe and capable of inducing specific immune responses against these unique tumor antigens [103,104,105,106,107]. These vaccines are designed based on individual tumor mutations identified through advanced sequencing technologies.

Ongoing clinical trials are testing various vaccine platforms, including dendritic cells, viral vectors, RNA, and peptides [103,104,105,106,107]. The aim is to evaluate the clinical effectiveness of these vaccines, especially in combination with other immunotherapies such as ICIs. This combination strategy is expected to enhance the immune system’s response to melanoma.

#### 4.5.2. Adoptive Cell Transfer

Adoptive cell transfer, especially tumor-infiltrating lymphocyte (TIL) therapy, has emerged as a noteworthy strategy in the management of melanoma, offering a personalized approach to cancer treatment. TIL therapy involves extracting immune cells from a patient’s tumor, expanding and enhancing these cells in a laboratory, and then reintroducing them into the patient to bolster the immune system’s ability to fight cancer. This method leverages the unique ability of TILs to recognize and target tumor-specific antigens, making it a potent form of immunotherapy [108,109,110].

Clinical trials have underscored the efficacy of TIL therapy in patients with metastatic melanoma, particularly those unresponsive to conventional treatments. Approximately 50% of patients achieved a partial response, while around 20% achieved a complete response, showcasing the potential of TIL therapy as a personalized form of immunotherapy [108,111]. Furthermore, patients treated with TIL therapy demonstrated an increase in median OS by 6 to 12 months compared to patients receiving standard treatments, highlighting the promise of TIL therapy in extending the lives of patients with advanced melanoma.

However, challenges remain, including the variability in response rates among patients and the logistical complexities involved in cell extraction and expansion. Moreover, the therapy’s side effects, such as cytokine release syndrome, necessitate careful patient monitoring [112]. Future research is directed towards enhancing the efficacy of TIL therapy through combination treatments, improving patient selection criteria, and understanding the genomic correlates of response to therapy [113,114].

#### 4.5.3. Microbiome and Melanoma Treatment Interactions

Recent research has brought to light the important role of the gut microbiome in the development and treatment of melanoma [115]. The gut microbiome, a complex ecosystem of microorganisms located primarily in the intestinal tract, influences various aspects of health, including immune system regulation and inflammation prevention [116,117]. 

The skin microbiome’s involvement in melanoma is gaining attention, with specific bacterial compositions linked to melanoma, such as an abundance of *Fusobacterium* and *Trueperella* genera [118]. Additionally, certain bacteria like *Corynebacterium* are associated with acral melanoma progression [119]. The presence of *Staphylococcus aureus* in other skin cancers suggests broader implications of skin microbiota in skin pathologies [120]. Probiotic and prebiotic applications show potential in mitigating UVR-induced skin damage and modulating the tumor immune microenvironment, indicating a promising, but yet to be clinically validated, approach to melanoma treatment and prevention [120,121]. 

The interplay between genetic mutations and the microbiome is highlighted in melanomagenesis, with *BRAF* mutations possibly influencing gut microbiota composition. This relationship, explored in colorectal cancer, suggests the potential for microbiota profiles as biomarkers and therapeutic targets in *BRAF*-mutated cancers, though evidence in melanoma is limited [122,123].

Immunotherapy has revolutionized melanoma treatment, with research focusing on microbiota’s role in modulating responses to ICIs [124,125,126]. Specific gut bacteria like *Akkermansia muciniphila* and *Bifidobacterium* species are associated with positive ICI responses, with studies exploring fecal microbiota transplantation to enhance immunotherapy efficacy [124,125,126]. Conversely, the microbiome may influence ICI-related adverse effects, with certain microbial profiles linked to reduced toxicity, suggesting the potential to tailor treatment and manage side effects through microbiota manipulation [127,128].

Ongoing clinical trials in the UK and the US are investigating the relationship between gut microbiota and melanoma treatment outcomes, focusing on immunotherapy efficacy and side effects [97,98,99]. Additionally, the field of molecular pathological epidemiology (MPE) integrates various disciplines to understand tumor–environment–host interactions, offering insights into melanoma pathogenesis and treatment [100,101,102,103]. Challenges such as validating molecular assays and sample size limitations persist [103].

Clinical trials in the UK and the US are investigating the relationship between gut microbiota and melanoma treatment, with a focus on immunotherapy efficacy and side effects. In the UK, one study is assessing gut microbiota diversity’s impact on immunotherapy outcomes and adverse reactions in stage 3 and 4 melanoma patients [129]. Another initiative aims to determine gut microbiota’s potential as biomarkers for immunotherapy effectiveness and toxicity in patients with unresectable stage 3 and 4 melanoma [130]. Meanwhile, in the US, researchers are exploring the effectiveness of fecal microbiota transplants combined with Pembrolizumab for treating PD-1 resistant/refractory melanoma, representing a significant advancement in melanoma treatment [131]. 

The interaction between lifestyle, environmental factors, diet, and other external influences on the genomic and metabolomic profiles of cells, including immune cells, is increasingly recognized [132]. Molecular pathological epidemiology (MPE) integrates epidemiology, bioinformatics, and biostatistics to understand tumor–environment–host interactions comprehensively [133]. Leveraging cutting-edge technologies like in vivo pathology and artificial intelligence, MPE investigates exogenous factors’ role in gut microbiota and their influence on melanoma pathogenesis and treatment [134]. This integrative approach facilitates exploring etiologic heterogeneity across diseases and establishing causal links between environmental factors and molecular biomarkers [135].

#### 4.5.4. Nanoparticle-Based Combination Therapy for Melanoma

Nanotechnology in melanoma treatment uses nanoparticles (NPs) to target drug delivery to cancer cells. This approach is significant in cancer treatment and also extends to areas such as diagnosis, gene therapy, biomarker creation, targeted therapy, and imaging. The biological properties of NPs allow them to accurately target cancer cells while minimizing the effects on healthy tissues [136].

In chemotherapy, NPs have been explored for their potential to deliver chemotherapeutic agents directly to melanoma cells. This method reduces systemic toxicity and degradation of the drugs. Specifically, carbon nanotubes have been utilized to deliver doxorubicin, a chemotherapeutic agent, directly to melanoma cells [137]. This approach has shown impressive results, with doxorubicin-loaded carbon nanotubes increasing cell death by 90% through inducing a moderate G2-M phase arrest (17.7 ± 1.1%) and significantly reducing tumor size in mice bearing B16–F10 melanoma [137]. Another approach involves the use of chitosan/alginate NPs for the delivery of doxorubicin. This method has been explored for its ability to control the release rate of doxorubicin, achieving better transport and higher intracellular concentration in melanoma cells [138]. The sustained release provided by chitosan/alginate nanoparticles leads to improved accumulation and prolonged cytotoxic effects of encapsulated doxorubicin in melanoma cell lines, showcasing the versatility and potential of nanoparticle-based delivery systems in improving chemotherapy outcomes.

In immunotherapy, nanovaccines like the DGBA-OVA-CpG (Guanidinobenzoic acid-ovalbumin-cytosine-guanine dinucleotides) nanovaccine have shown potential in increasing the generation of cytotoxic T-lymphocytes and reducing melanoma growth when combined with checkpoint inhibitors [139]. This underscores the ability of nanoparticles to modulate the tumor microenvironment and overcome immunosuppression. 

In PDT, NPs improve the delivery and efficacy of photosensitizers. NPs increase PSs’ solubility, permeability, and retention in targeted cells, facilitating deeper penetration and reducing treatment resistance by generating high levels of reactive oxygen species (ROS). Studies have shown that NPs can be used to deliver 5-aminolevulinic acid (5-ALA) and other PSs, like phthalocyanine 4 encapsulated in silica NPs, leading to increased apoptosis and reduced tumor survival in melanoma cell lines [140,141]. Additionally, novel approaches like yttrium oxide NPs combined with X-rays have shown potential in increasing ROS production and causing DNA damage, indicating a promising direction for enhancing PDT’s efficacy [142]. 

Nanoparticle-based therapies offer a promising approach to melanoma treatment, aiming for more precise and effective strategies with minimal harm to healthy tissue. Ongoing research and clinical trials are crucial for moving these innovations from the lab to practical use, ultimately enhancing treatment outcomes for melanoma patients.

#### 4.5.5. DNA Damage Response Inhibitors

Melanoma is characterized by its high mutational burden and frequent mutations in DDR genes, leading to increased DNA damage and replicative stress [143]. DDR inhibitors, including inhibitors of DNA-PKcs, PARP, ATM, CHK1, WEE1, and ATR, have demonstrated promising results in both preclinical and clinical studies to enhance the efficacy of existing therapies, including chemotherapy, targeted therapy, and immunotherapy [143].

For instance, PARP inhibitors, which act through mechanisms like the inhibition of PARP function and “trapping” PARPs on DNA lesions, have shown potential in reducing migration and invasion and inducing apoptosis in melanoma cell lines [144,145,146]. Clinical trials combining PARP inhibitors with chemotherapy, particularly temozolomide, have shown improvement in progression-free survival (PFS), albeit without reaching statistical significance [147,148]. 

Targeting WEE1 has been shown to increase DNA damage and cell death in melanoma cell lines, suggesting its potential as a therapeutic target [149]. The small molecule inhibitor adavosertib, targeting WEE1, has demonstrated cytotoxic effects alone and in combination with other treatments in melanoma, highlighting its potential for clinical application [150,151,152,153]. 

The combination of ATR inhibitors with PARP inhibitors, specifically AZD-6738 and olaparib, has been found to effectively target *BRAF*V600 mutant melanoma cell lines that have developed either primary or acquired resistance to BRAF and MEK inhibitors, demonstrating a high susceptibility to this treatment strategy [154]. Clinical trials, such as those involving ceralasertib, have demonstrated encouraging results, particularly in melanomas resistant to PD-1 inhibitors, suggesting the value of ATR inhibitors in enhancing immune responses and improving outcomes in melanoma treatment [155,156].

Targeting DNA-PKCS, a key component in the DNA repair process, has emerged as a promising strategy for combating resistance to MAPK inhibitors (MAPKi) in melanoma.

In human melanoma cell lines and patient-derived xenografts (PDX), nonhomologous end-joining (NHEJ) targeting by a DNA-PKCS inhibitor prevents/delays acquired MAPKi resistance by reducing the size of ecDNAs and CGRs early in combination treatment [157].

In conclusion, DDR inhibitors represent a promising therapeutic avenue for melanoma treatment, potentially enhancing the effectiveness of current therapies and overcoming resistance. Ongoing and future clinical trials will be crucial in determining their role in melanoma management.

#### 4.5.6. LAG-3 Inhibitors 

LAG-3 (lymphocyte-activation gene 3) inhibitors have become a significant development in melanoma immunotherapy, especially when used in combination with PD-1 inhibitors. These inhibitors, now FDA-approved as first-line therapy for metastatic melanoma, demonstrate considerable efficacy and manageable toxicity levels [158]. LAG-3 operates by attaching to MHC class II molecules on antigen-presenting cells, which suppresses TCR signaling and T cell activation, playing a crucial role in the tumor microenvironment to aid melanoma cells in evading immune detection [158]. 

The collaboration of relatlimab, an anti-LAG-3 antibody, with nivolumab, a PD-1 inhibitor, represents a significant step forward in the treatment of melanoma. Approved in 2022 for treating metastatic or unresectable melanoma, this duo therapy has led to superior PFS outcomes, particularly highlighted in the RELATIVITY-047 trial [159]. This trial showed a PFS of 10.1 months for the combined therapy, as opposed to 4.6 months for nivolumab alone at a median follow-up of 13.2 months, showcasing the effectiveness of LAG-3 inhibition alongside PD-1 blockade [160].

**Table 2:** Comprehensive overview of melanoma therapies: from traditional approaches to emerging treatments.

Therapy	Mechanism of Action	Clinical Outcomes	Adverse Effects	References
Targeted therapy (BRAF inhibitors)	Disrupts MAPK/ERK signaling pathway by targeting mutated BRAF protein	Improved PFS and OS in *BRAF*-mutated melanoma	Skin rash, headache, fever, joint pain	[63,64,65,66]
Targeted therapy (MEK inhibitors)	Targets MEK1 and MEK2 downstream of BRAF in MAPK signaling pathway	Enhanced efficacy in metastatic melanomas	Fatigue, rash, diarrhea, hypertension	[68,69,70,71]
BRAF-MEK combination therapy	Reduces resistance development by blocking multiple points in the MAPK pathway	Increased PFS and a higher overall response rate compared with monotherapy	Increased liver enzymes, fever, fatigue, dermatitis	[72,73,74,75,76]
Checkpoint inhibitors: PD-1 and CTLA-4	Enhances immune system’s ability to recognize and attack cancer cells by blocking PD-1 receptor /by blocking immunosuppressive interaction between CTLA-4 and B7	Significant survival advantage in advanced melanoma, though associated with high toxicity levels	Fatigue, skin rash, pruritus, colitis	[78,79,80,81,82,83,84]
Talimogene laherparepvec (T-VEC)	Genetically modified herpes simplex virus type 1 induces local and systemic immune responses	Effective as monotherapy and in combination with ICIs, in unresectable metastatic stage IIIB/C–IVM1a melanoma melanomas	Flu-like symptoms, fatigue, chills, fever	[87,88,89,90,91,92,93,94]
Combination of targeted therapy and immunotherapy	Combines targeted therapy’s direct action on genetic mutations with immunotherapy’s broad-acting capacity	Provides clinically significant survival benefits in patients with *BRAF*-mutant melanoma	Varies based on combination	[95,96,97]
Integration of surgical and systemic therapies	Combines surgical interventions with systemic therapies to improve outcomes	Improves OS rates, reduces recurrence, and manages metastatic melanoma more effectively	Dependent on specific therapies used, varies	[67,98,99,100,101,102]
Neoantigen vaccines	Tailored to individual patients targeting unique tumor-specific antigens from mutations	Safe, induces specific immune responses against unique tumor antigens	Injection site reactions, flu-like symptoms	[103,104,105,106,107]
Adoptive cell transfer (TIL therapy)	Uses patient’s own T cells, expanded and enhanced in lab, then reintroduced to patient to fight cancer	Improves OS in advanced melanoma	Cytokine release syndrome, requires monitoring	[108,109,110,111,112,113,114,161,162]
Microbiome and melanoma treatment interactions	Gut microbiome’s role in treatment responses, leading to new strategies	Influences outcomes and adverse reactions of immunotherapy	Varies	[115,116,117,118,119,120,121,122,123,124,125,126,127,128,129,130,131,132,133,134,135]
Nanoparticle-based combination therapy	Uses nanoparticles for targeted drug delivery to cancer cells, reducing effects on healthy tissues	Improves chemotherapy outcomes and enhances PDT efficacy	Varies, specific to nanoparticle type	[136,137,138,139,140,141,142]
DNA damage response inhibitors	Targets DNA damage response (DDR) genes	Enhances efficacy of existing therapies, including chemotherapy, targeted therapy, and immunotherapy	Anemia, nausea, fatigue, neutropenia	[143,144,145,146,147,148,149,150,151,152,153,154,155,156,157]
LAG-3 inhibitors	Targets LAG-3 to enhance immune response	Superior PFS outcomes when used with PD-1 inhibitors in metastatic or unresectable melanoma	Fatigue, diarrhea, pruritus, rash	[158,159,160]

## 5. Discussion and Conclusions

Melanoma treatment has seen dramatic advancements with the introduction of BRAF and MEK inhibitors, steering away from conventional chemotherapy, such as dacarbazine, towards a more personalized medicine approach. This pivotal shift has facilitated the emergence of immunotherapies, notably pembrolizumab and nivolumab, offering renewed hope for patients with advanced stages of the disease. The evolution towards targeted treatments and immunotherapies represents a significant leap in melanoma care, reflecting a deeper understanding of its molecular underpinnings.

Despite these advancements, the journey is far from over. Challenges such as resistance to targeted therapies and the variable efficacy of immunotherapies persist, underscoring the complexity of melanoma treatment. High costs and limited accessibility further complicate the delivery of these advanced treatments, particularly in resource-poor settings. The management of in-transit metastases also presents an area requiring more targeted research efforts, indicating gaps within current treatment paradigms.

The path forward is multifaceted, highlighting the importance of ongoing research into resistance mechanisms, the development of predictive biomarkers, and the creation of cost-effective care strategies on a global scale. Novel approaches to treatment strategies, such as personalized neoantigen vaccines, adoptive cell transfer, and the exploration of the microbiome’s role in melanoma treatment, underscore the dynamic nature of melanoma research and therapy. These innovative strategies offer the promise of further personalizing cancer care, enhancing the immune system’s response to melanoma, and overcoming the limitations of current treatments.

Reflecting on these innovative approaches reinforces the critical need for integrating emerging therapies into clinical practice. The exploration of treatments such as nanoparticle-based therapies and DNA damage response inhibitors represents a promising frontier in overcoming resistance to current therapies, potentially heralding a shift in how melanoma is treated on a molecular level. Furthermore, the collaboration of LAG-3 inhibitors with PD-1 inhibitors represents a significant advancement in immunotherapy, offering improved outcomes for patients.

In conclusion, while significant progress has been made in understanding and treating melanoma, translating these findings into widespread clinical practice is essential. The commitment to overcoming existing challenges, ensuring equitable access, and focusing on long-term outcomes remains paramount. Future research must continue to evolve, integrating novel approaches to treatment strategies to refine treatment approaches and, ultimately, achieve more personalized and effective care for all melanoma patients. 

## Figures and Tables

**Table 1:** Molecular Biomarkers in Melanoma.

Biomarker	Comments	References
*BRAF*V600E Mutation	Responsive to BRAF inhibitors. Indicates early diagnosis, staging, and prediction of therapy responses.	[41,42,43]
*NRAS* Mutation	Linked to shorter survival times in stage IV melanoma. Less common, but significant.	[44,45,46]
*c-KIT* Mutation	Not closely associated with histological subtypes or tumor stage. Prevalent in older patients, acral mucosal melanoma, and sun-damaged areas.	[47,48,49,50,51]
*NF1* Mutation	Linked to a poorer prognosis than other mutation patterns.	[52,53]
PMCA4 Transcript Levels	High levels in females are associated with longer progression-free survival (PFS) and improved prognosis, especially following PD-1 blockade therapy.	[54]
Tumor Mutational Burden (TMB)	High TMB might correlate with increased effectiveness of immune checkpoint inhibitor (ICI) treatments.	[55,56,57]
Gene Expression Profiling (GEP)	GEP tests in melanoma provide prognostic data on recurrence and metastasis risk.	[58,59]
Gut Microbiome	Intestinal microbiota composition affects response to ICI therapy. Greater diversity, particularly *Ruminococcaceae* subspecies, is associated with better outcomes.	[60,61]
